# Discrete roles of canonical and non-canonical Wnt signaling in hematopoiesis and lymphopoiesis

**DOI:** 10.1038/cddis.2015.326

**Published:** 2015-11-19

**Authors:** F Famili, B A E Naber, S Vloemans, E F E de Haas, M M Tiemessen, F J T Staal

**Affiliations:** 1 Department of Immunohematology and Blood Transfusion (IHB), Leiden University Medical Center, Leiden, The Netherlands

## Abstract

The mechanisms that regulate proliferation, fate decisions and differentiation of hematopoietic stem cells (HSC) and thymic stem cells are highly complex. Several signaling pathways including Wnt signaling have important roles during these processes. Both canonical and non-canonical Wnt signaling are important in normal and malignant hematopoiesis and lymphoid development, yet their precise roles are controversial. In a side-by-side comparison, we investigated the roles of the canonical and non-canonical Wnt pathway in hematopoiesis and thymopoiesis. As complete loss-of-function models for non-canonical Wnt signaling are not yet available and highly complex for canonical Wnt signaling, we decided to use a gain-of-function approach. To this end, Wnt3a and Wn5a, two well-known prototypical canonical and non-canonical Wnt ligands were produced in hematopoiesis supporting stromal assays. High levels of Wnt3a signaling blocked T-cell development at early stages, whereas intermediate levels accelerated T-cell development. In contrast, Wnt5a signaling prompted apoptosis in developing thymocytes, without affecting differentiation at a particular stage. To explore the role of Wnt3a and Wnt5a *in vivo*, we transduced HSCs isolated from fetal liver, transduced with Wnt3a and Wnt5a vectors, and performed reconstitution assays in irradiated C57Bl/6 mice. Wnt3a overexpression led to increased lymphopoiesis, whereas Wnt5a augments myelopoiesis in the bone marrow (BM) and spleen. Thus, the canonical and non-canonical Wnt signaling have discrete roles in hematopoiesis and thymopoiesis, and understanding their right dose of action is crucial for prospective translational applications.

The development of blood and immune cells are highly complex and regulated processes. A wide variety of signaling pathways has been implicated in these processes. Several developmental signals have key roles in both the bone marrow (BM) and thymus, such as BMP, Wnt and Notch signaling.^1, 2^ Hematopoietic stem cells (HSC) are rare BM-residing cells with the capacity to self-renew and differentiate into all blood cell lineages. All blood cells, except T lymphocytes, develop within the BM. Different types of progenitor cells migrate from BM to thymus where they develop to mature T cells.^3, 4^ The nature of these cells is still subject of debate. As only few early thymic progenitors (ETPs) arrive in the thymus (<10/day), massive proliferation is necessary to establish a pool of T-cell progenitors.^5^ During development, these immature thymocytes gradually lose their proliferative and multilineage potential, and initiate a T-cell developmental program, a process termed T-cell commitment. Notch signaling has been shown to have an important role during T-cell commitment by directly or indirectly upregulation of T-cell-specific genes including *Ptcra*, *Cd3e* and *Zap 70*.^6, 7^ Other soluble factors including Wnt ligands might also be crucial for T-cell proliferation and commitment.^8, 9, 10^

Early stages of T-cell development are phenotypically characterized by absence of the mature T-cell markers CD4 and CD8 and referred to as double negative (DN).^11^ DN stages are subdivided into four stages. DN1: CD44+ CD25–, DN2: CD44+ CD25+, DN3: CD44− CD25+ and DN4: CD44– CD25–.^8, 12^ It is believed that T-cell commitment occurs at the transition of DN2 to DN3 stages.^13, 14^ Afterwards, thymocytes develop to the immature single positive (ISP) stage defined as CD3− CD8+. Thymocytes with functionally rearranged T-cell receptors (TCRs) develop to next stage, which is double positive (DP) for CD4 and CD8 and finally they become mature single positives (SP)^12^ either CD4 or CD8.^7, 8, 9^

Thymic epithelial cells (TECs) provide a unique environment for ETPs to develop towards T cells.^15, 16^ TECs also express high levels of Notch ligands including Delta like ligands 1 and 4, soluble Wnt ligands and IL-7, which all are crucial for early stages of T-cell development.^17^

The Wnt signaling pathway is subdivided into canonical (*β*-catenin dependent) and non-canonical (*β*-catenin independent) pathways. Binding of different Wnt proteins to frizzled (Fzd) receptors can trigger different Wnt pathways. The diversity of ligands and receptors makes the study of Wnt signaling from point of view of cell surface receptors and ligands challenging. Wnt proteins function as proliferation-inducing growth factors but may also affect cell-fate decisions, apoptosis and quiescence.^18^ Canonical Wnt proteins bind to their receptors, thereby preventing proteosomal degradation of the Wnt-mediator *β* -catenin. Subsequently, *β* -catenin trans locates to the nucleus where it will form an active transcription complex with one of the four transcription factors downstream of the Wnt pathway: Tcf1, 3 or 4 (T-cell Factor 1, 3, 4) or Lef1 (lymphocyte-enhancer-binding factor). Upon transcriptional activation, several target genes will be activated including *Axin2, c-fos, c-myc* and many others, which are important for proliferation and/or cell-fate decisions. Non-canonical Wnt signaling involves recognition of distinct Wnt ligands by a cognate Frz-LRP receptor complex, heterotrimeric G protein activation of phospholipase C as well as the release of intracellular Ca^2+^ ions. Non-canonical Wnt signaling also regulates cellular polarization and migration (the so-called planar-cell-polarity pathway).^9, 10, 18, 19, 20, 21^

A large body of evidence has shown the significance of canonical Wnt signaling during T-cell development. Generation of Tcf1 KO mice provided the first evidence of a Wnt signaling effect during T-cell development.^22^ Tcf1 deficiency partially blocks T-cell development at various early DN stages, resulting in fewer mature T cells and smaller thymus. In addition, Tcf1/Lef double KO mice have a complete block at the ISP stage, which indicates redundancy between these factors during thymocyte development.^23^

Similar to Tcf1 deficiency, fetal thymic organ cultures using Wnt3a-deficient progenitors exhibited progressively impaired T-cell development caused to an ISP block.^24^ Several loss-of-function and gain-of-function studies have targeted the core Wnt-mediator *β*-catenin in thymocytes. Conditional *β*-catenin deletion using Lck-Cre impaired *β*-selection of TCR,^25^ whereas *β*-catenin overexpression regulates positive selection and generation of SP CD4/ CD8.^26^ Inhibiting the interaction between *β*-catenin and Tcf1 also blocks the DN to DP transition.^27^ Wnt signaling is active at various stages of T-cell development but most predominantly at DN stages. Indeed, inhibiting Wnt signaling by using Dickkopf (DKK1) as Wnt sequestering molecule, blocks the development at the most immature DN1 stage.^21^ Finally, genetic proof that canonical Wnt signaling is crucial for normal T-cell development stems from complementation studies in which only the large form of Tcf1 that can interact with *β*-catenin and transduce Wnt signals was capable to restore T-cell development, whereas the short form that lack the *β*-catenin domain could not.^28^

On contrary, there are only a handful of studies focused on the role of non-canonical Wnt signaling in T-cell development. Liang *et al.*^29^ showed that Wnt5a deficiency protects against apoptosis in DP stage, but it was proposed to be irrelevant at early stages of T-cell development. Another series of studies revealed that Wnt4 induces expansion of Lin- Sca1+ Kit+ (LSKs) in BM, which subsequently causes ETP expansion in thymus.^30^ Wnt4 regulates ETP expansion via a TEC-dependent mechanism.^31^ Whether Wnt4 functions as a canonical or non-canonical ligand is still debatable, in particular in gain-of-function studies in which thymopoiesis was increased. Perreault and colleagues showed that Wnt4 binds to Fzd6 and activates JNK kinase via PCP pathway,^32^ strongly suggesting that Wnt4 signals in a non-canonical fashion.

Loss-of-function approaches using *β*-catenin and *γ*-catenin have often not revealed thymic phenotypes,^33^ most likely because Wnt signaling is still present at appreciably levels in these models.^34^ In addition, during T-cell development canonical and non-canonical Wnt signaling have only been studied in isolation but not together. Here, we aimed to side-by-side compare the two prototypical canonical and non-canonical Wnt ligands, that is, Wnt3a (canonical) and Wnt5a (non-canonical).

## Results

### OP9-based *in vitro* assays for T-cell development

**Figure 1:**
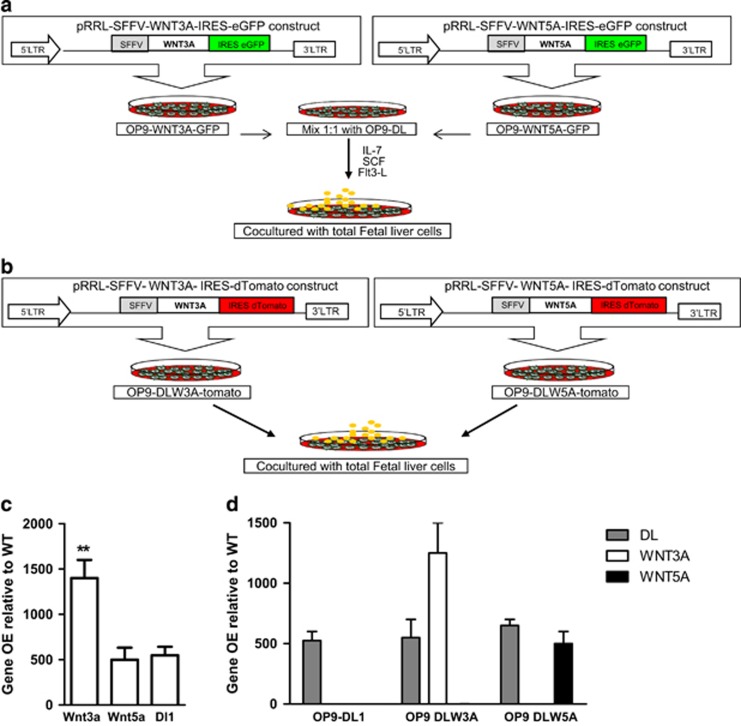

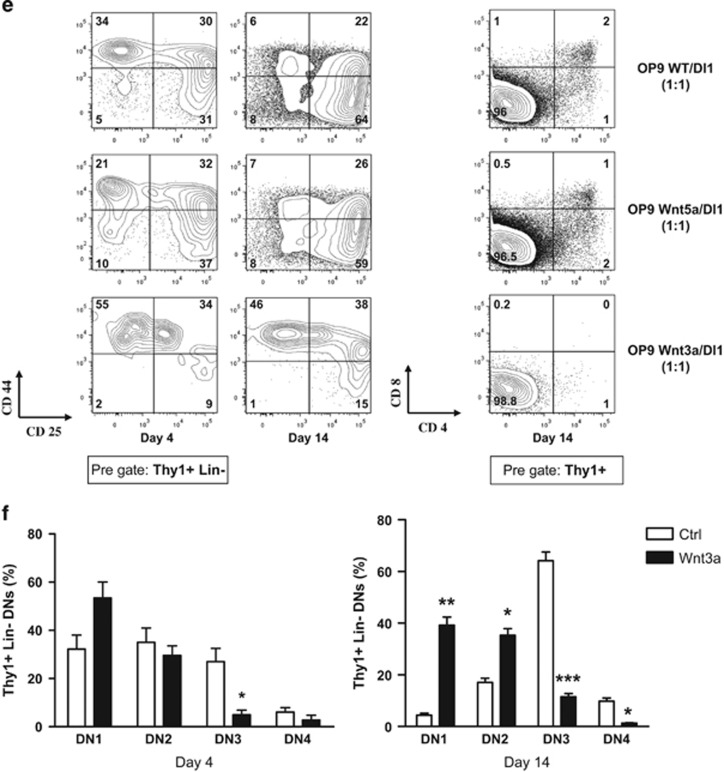
**C**anonical Wnt3a overexpression blocks T-cell development at early stages. (**a**) Experimental design. OP9-WT cell lines were transduced with pRRL-SFFV-Wnt3a-GFP or pRRL-SFFV-Wnt5a-GFP lentiviral constructs, selected by cell sorting on high GFP expression and for experiments mixed with OP9-Dl1-GFP. Stem cells from E14 fetal liver cells were co-cultured with OP9-WT/Dl1 mixed 1:1 as a control, or OP9-Wnt3a/Dl1 1:1, or OP9-Wnt5a/Dl1 1 : 1 for 14–21 days in aMEM 10% FCS in presence of IL-7, SCF and Flt3 L cytokines. (**b**) Experimental design. OP9-Dl1-GFP cell lines were transduced with pRRL-SFFV-Wnt3a-tomato (DLW3A) or pRRL-SFFV-Wnt5a-Tomato (DLW5A) and selected by cell sorting on high GFP expression. Stem cells from E14 fetal liver cells were co-cultured with OP9-Dl1 as a control, OP9-DLW3A or OP9-DLW5A for 14 days in aMEM 10% FCS in presence of same cytokines. (**c**) RTq-PCR analysis was performed to determine overexpression of *Wnt3a* (left bar), *Wnt5a* (middle bar) and *Dl1* gene (right bar) in transduced OP9 cell lines. The levels of expression are normalized by *ABL-2* expression and presented as fold induction relative to untransduced OP9 cell line. (**d**) The overexpression of *Dl1* gene (gray bars), *Wnt3a* gene (white bars) and *Wnt5a* gene (black bars) in untransduced OP9DL1 cell line (left), OP9-DLW3A cell line (middle) and OP9-DLW5A (right) are shown. The levels of expression are normalized by *ABL-2* expression and presented as fold induction relative to untransduced OP9 cell line. (**e**) Total fetal liver cells were co-cultured with OP9-WT/DL1 : 1 as control (top row), OP9-Wnt5a/DL1:1 (middle row) or OP9-Wnt3a/DL1 : 1 (bottom row). Cells were harvested 4 days and 14 days after co-culture and were analyzed flow cytometric for DN stages of T-cell development. The plots are pre-gated for Thy1+ and LIN- markers. Lineage markers include CD3e, CD4, CD8a, CD11b, Gr1, B220, NK1.1 and Ter119. The percentage of each population is indicated. (**f**) Collective data of total experiments from (**e**) are depicted. Total fetal liver cells are co-cultured with OP9-WT/DL1 : 1 as control (white bars) or with OP9-Wnt3a/DL11 : 1 (Black bars). Cells were harvested at day 4 and day 14 of co-culture and were assessed by FACS for DN stages of T-cell development. The percentage of each stage is shown within Thy1+ Lin– population. Data are mean±S.D. of nine control and eight Wnt3a samples from three independent experiments. *P<0.05; **P<0.01; ***P<0.001

To study the role of canonical and non-canonical Wnt signaling during T-cell development *in vitro*, we generated two different OP9-based assays. First, we transduced OP9-WT cell line with Wnt3a and Wn5a constructs linked to an IRES-GFP cassette, mixed the transduced cell lines with the OP9-Dl1 cell line in 1:1 ratio to support T-cell development (Figure 1a). Initially, we examined efficiency of the OP9 cell mixtures for support of T-cell development in comparison with OP9-Dl1 cell line alone. The aim of mixing Wnt3a- and Wnt5a-producing OP9 cells with OP9-DL11 was (a) supporting T-cell development in contrasting situation of abundant Wnt3a vs Wnt5a (b) creating an culture system to study T and B lymphoid as well as myeloid development simultaneously. Although there was a slight delay in T-cell development at 14–21 days, the mixture could also efficiently support T-cell development up to the DP stages, whereas OP9-WT did not induce T-cell development (See Supplementary Figure 1).

We also generated another OP9-based assay in which we directly transduced OP9- Dl1-GFP cell line with Wnt3a and Wnt5a-tomato constructs, and assessed *in vitro* T-cell development (Figure 1b). We quantified relative expression of Wnt3a, Wnt5a and Dl1 using Q-PCR. Wnt3a had over 1000-fold higher expression compared with OP9-WT, whereas Wnt5a had ~500-fold and Dl1 remained to be expressed at levels over 700-fold (Figure 1c). In OP9-DLWnt3a-tomato (DLW3A) and OP9-DLWnt5a-tomato (DLW5A) Wnt3a and Wnt5a were expressed as high as in the OP9-Wnt3a-GFP and OP9-Wnt5a-GFP, respectively, and Dl1 expression was not altered compared with the OP9-Dl1-GFP (Figure 1d).

### Canonical Wnt3a overexpression blocks T-cell development at early stages, thereby favoring development of alternative lineages

We cultured fetal liver cells (as these provide a good source of both T-cell progenitors and stem cells) either with the mixture of OP9-WT/Dl1 in 1:1 ratio as a control, or with OP9-Wnt3a/Dl1 or OP9-Wnt5a/Dl1 with the same ratio for 14 days. Wnt3a overexpression blocked T-cell development at the DN1 stage (Figures 1e and f).

**Figure 2:**
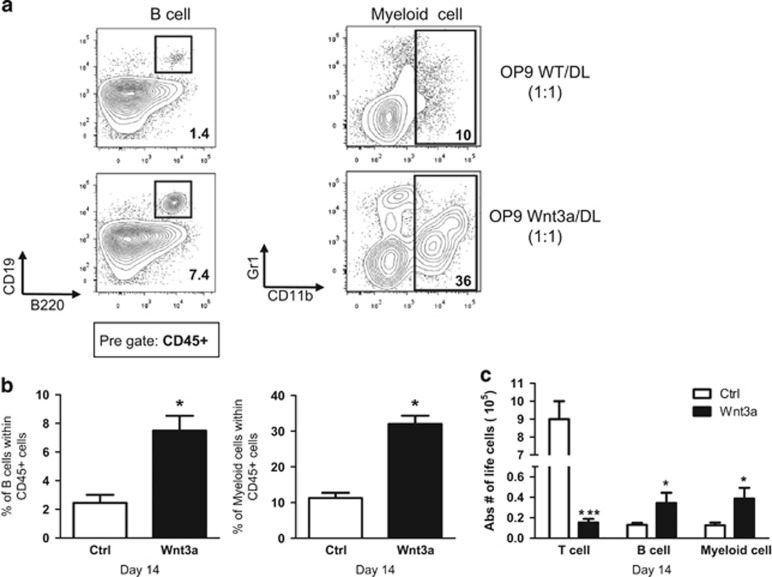
Wnt3a overexpression favors alternative lineages development. (**a**) Total fetal liver cells were co-cultured with OP9-WT/DL1 : 1 (top row) or with OP9-Wnt3a/DL11 : 1 (bottom row). Cells were harvested after 14 days of co-culture and were analyzed by FACS for B cells (B220 and CD19) and myeloid cells (CD11b and Gr1). The plots are pre-gated on CD45+ cells. Representative plots of two independent experiments are shown. (**b**) Collective data of all experiments are depicted. OP9-WT/DL (Ctrl) are depicted in white bars and OP9-Wn3a/Dl (Wnt3a) in black bars. The left bar graphs present percentage of B cells (B220+ CD19+) within the CD45+ gate, and right bar graphs present percentage of myeloid cells (CD11b+ Gr1+) within the CD45+ gate. (**c**) Absolute number of total CD45+ life cells after 14 days of co-culture are depicted. Data are mean±S.D. of six controls and five Wnt3a samples from two independent experiments. *P<0.05; **P<0.01; ***P<0.001

The mixture of OP9-WT and OP9-Dl1 provides a means to study development of B, T, NK and myeloid lineages simultaneously.^35^ DN1 and DN2 immature thymocytes have the potential to develop towards other lineages. Consistently, in the Wnt3a-overexpressing cultures, non T cells, such as B and myeloid cells developed more efficiently compared with the control (Figures 2a and b). The difference was not only observed in proportion of each lineage, but also in the absolute numbers (Figure 2c).

Similar data were observed when Wnt3a and Wnt5a were expressed in the OP9-Dl1 cells themselves (supplementary Figure 2). Therefore, we conclude that canonical Wnt3a signaling has the capacity to inhibit T-cell development at early DN2-DN3 stages, and to increase alternative (myeloid and B cell) development. Wnt5a overexpression in neither of assays did show any phenotypic difference regarding T-cell development (data not shown).

### Wnt5a overexpression results in increased apoptosis in developing thymocytes, whereas Wnt3a does not

**Figure 3:**
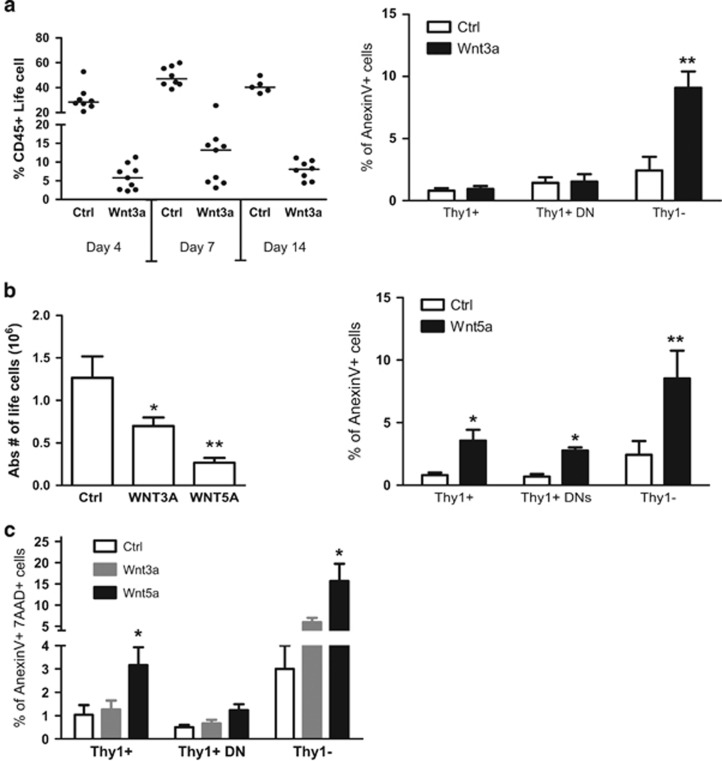
Wnt5a overexpression results in apoptosis in developing thymocytes. (**a**) Total fetal liver cells were co-cultured with OP9-WT/DL1 : 1 (Ctrl) or with OP9-Wnt3a/DL1 : 1 (Wnt3a) or with OP Wnt5a/Dl1 (Wnt5a). In the left graph, the percentage of total CD45+ life cells were assessed by FACS after 4 days, 7 days and 14 days of co-culture. The averages are indicated by a dash. Each dot represents one mouse. In the right graph, the percentage of AnexinV+ apoptotic cells is shown in specified populations after 14 days of co-culture with OP9-WT/DL (white bars) or with OP9-Wnt3a/DL (black bars). Error bars represent mean±S.D. from two independent experiments (each in triplicates). (**b**) In the left graph, the absolute number of life cells is shown after 14 days of co-culture. Error bars represent mean±S.D. from two independent experiments (each in triplicates). In the right graph, the percentage of AnexinV+ apoptotic cells is shown in specified populations after 14 days of co-culture with OP9-WT/DL (white bars) or with OP9-Wnt5a/DL (black bars). Error bars represent mean±S.D. from two independent experiments each in triplicates. (**c**) Percentage of AnexinV+AAD7+ cells representing dead cells in the culture gated on tall thymocytes, DN thymocytes and non-T lineage cells in thymic cultures with Wnt3a or Wnt5a expressed in OP9-DL1 cells. *P<0.05; **P<0.01; ***P<0.001

Further analysis revealed that total percentage of lymphocytes was around fourfold lower in the Wnt3a-overexpressing group at various time points after co-culture (4, 7 and 14 days) (Figure 3a). This could be caused by increased apoptosis in the Wnt3a-expressing cultures. We performed apoptosis assays by using AnexinV and 7AAD in combination with the Thy1 marker to separate T cells from non-T cells. In the Wnt3a-overexpressing cultures total Thy1+thymocytes and specifically DN thymocytes were not undergoing any a significant level of apoptosis (Figure 3a). There is an increase in apoptosis in non T cells, yet not to the extent that non T cells would decrease in numbers in comparison with thymocytes (see Figures 2b and c). Thus, we concluded that inhibition of differentiation toward T lineage due to Wnt3a overexpression causes low cellularity in this culture.

Although the Wnt5a-overexpressing cultures did not show any phenotypic differences compared with the controls, the absolute numbers were around three- to fivefold lower (Figure 3b). Lack of Wnt5a promotes Bcl-2 expression and inhibits apoptosis of DP thymocytes.^29^ Our analysis showed that both Thy1+ and Thy1– co-cultured cells were undergoing more apoptosis. Thus, this gain-of-function approach showing more apoptosis is consistent with the loss-of-function Wnt5a experiments reported before. The effect of apoptosis in thymocytes (DN and Thy1+) by Wnt5a was much stronger than Wnt3a, which was similar to controls. Interestingly, for non T cells, similarly high apoptosis was found. The strong effects of Wnt5a on cell death are also reflected in the strong increase in 7AAD+AnnexinV+ DP cells (Figure 3c).

### Optimal dosage of Wnt3a signaling accelerates T-cell development

Gene expression analysis revealed that Wnt3a was very highly expressed in our transduced OP9 cells (Figures 1c and d). This is 500-fold higher than the physiological level, for example, as found in fetal thymus,^21^ which we used as comparison. We have previously shown that canonical Wnt signaling functions in a dosage-dependent fashion during HSC reconstitution and T-cell development using Apc hypomorphic models.^19^ We hypothesized that the same would hold true with the Wnt3a gain-of-function model. We modified the Wnt3a concentration in the culture by serially mixing OP9-DL with OP9-DLW3a at various ratios.

**Figure 4:**
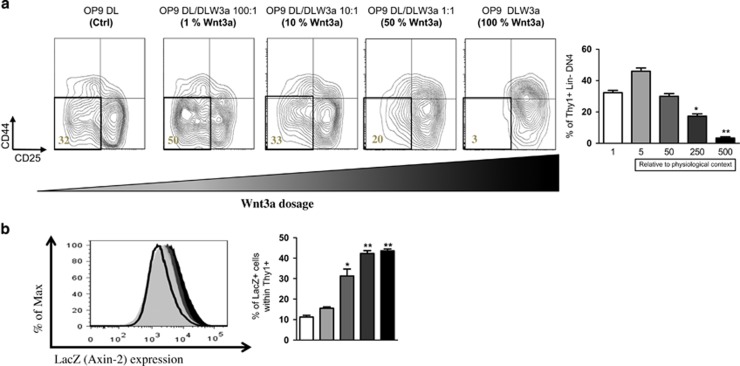
Optimal dosage of canonical Wnt signaling accelerates T-cell development. (**a**) Total FL cells were co-cultured for 14 days with OP9-DL1, or different ratios of OP9-DLW3A/ OP9-Dl1 mixture as indicated. Cells were then harvested and were analyzed by FACS for DN stages of T-cell development. The percentage of CD44− CD25− DN4 is indicated. The plots are pre-gated on Thy1+ LIN– markers. Error bars represent mean±S.D. of one independent experiment in triplicates. Below the bar graphs Wnt3a gene overexpression relative to the physiological levels are indicated. (**b**) Total FL cells from Axin2 LacZ (wnt reporter mice) were co-cultured with OP9-DL1 and different ratios of OP9-DLW3A as previously indicated. Cells were harvested and *β*-galactosidase (LacZ) activity in Thy1+ cells was measured. Quantification of the mean fluorescence intensity (MFI) of Thy1+ cells is shown (left). Quantification of the frequency of LacZ+ cells is shown (right). Littermate mice not carrying the reporter transgene (Axin2 +/+) were used to define the LacZ– population. Data represent four Axin2 +/− mice and two Axin2 +/+ control mice. Error bars represent mean±S.D. *P<0.05; **P<0.01; ***P<0.001

After 14 days of co-culture, the majority of cells developed into DN3 and DN4 with 32% of DN4 thymocytes. Interestingly, the co-cultured cells with 1% DLW3A (which has fivefold higher overexpression relative to the physiological level, supplementary Figure 3), showed an accelerated T-cell development with 50% DN4 thymocytes development. However, T-cell development was inhibited again by increasing the concentration of DLW3A (10% and 50% Wnt3a), and was completely blocked at the DN3 stage with 100% OP9-DLW3A alone (Figure 4a).

To determine the Wnt signaling activity, we performed the same experiment using Axin2^LacZ^ heterozygous reporter cells, a well-established Wnt reporter mouse model.^36^ Phenotypic analysis of the co-cultured cells exhibited similar T-cell development potential at different dosages of Wnt3a (data not shown). Importantly, the actual Wnt signaling activity of total Thy1+ cells correlated with the increasing concentration of Wnt3a (Figure 4b). Therefore, our data suggest that Wnt3a canonical Wnt signaling functions in a dosage-dependent fashion, in accordance with the differential Wnt signaling activity of the cells.

### Wnt3a overexpression enhances B lymphopoiesis and Wnt5a overexpression augments myelopoiesis *in vivo*

**Figure 5:**
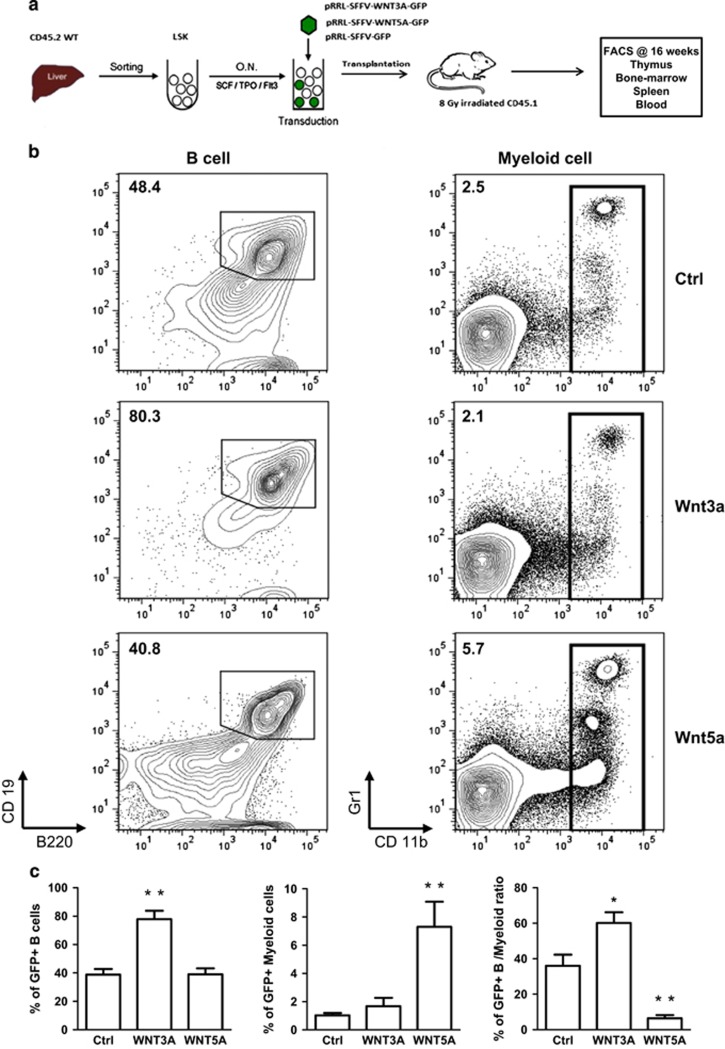
Wnt3a overexpression enhances lymphopoiesis, whereas Wnt5a overexpression augments myelopoiesis *in vivo*. (**a**) Experimental design. Lin– Sca1+ Kit+ (LSK) cells were sorted from E14 WT FL and were stimulated overnight in medium with SCF, TPO and Flt3-L cytokines. Next day, the cells were transduced with pRRL-SFFV-GFP (Ctrl), pRRL-SFFV-Wnt3a-GFP (Wnt3a) or pRRL-SFFV-Wnt5a-GFP (Wnt5a). Bulk of transduced cells were transplanted intravenously into CD45.1 8 Gy irradiated recipients. At week 16 post transplantation, the mice were killed and blood, spleen, BM and thymus were harvested and were assessed by FACS. Each group consists of five mice. (**b**) Representative FACS plots of B cell (B220+ CD19+) and myeloid cells (CD11b+ G1+) in the spleen of a recipient mouse 16 weeks after transplantation. The cells are pre-gated on CD45.2+ CD45.1− and GFP+. The numbers indicate percentage of cells within the gate. (**c**) Collective data represent the percentage of GFP+ B cells (left graph), myeloid cells (middle graph), and the ratio of B cells *versus* myeloid cells in the spleen of each group. Data are mean±S.D. of five mice per group. *P<0.05; **P<0.01; ***P<0.001

To study the role of Wnt3a and Wnt5a signaling *in vivo* we carried out reconstitution assays in which we sorted CD45.2 LSK cells from fetal liver, transduced them with lentiviral vectors encoding Wnt3a, Wnt5a in combination with GFP, and transplanted them into irradiated CD45.1 B6-recipient mice (Figure 5a). At week 16 post transplantation we analyzed spleen, thymus and BM of the recipients. In the spleens of Wnt3a-overexpressing mice the percentage of B220+ CD19+ B cells was around twofold higher compared with the control mice within the GFP+ transduced compartment. In contrast, Wnt5a-overexpressing mice had around twofold higher percentage of CD11b+ Gr1+ myeloid cells relative to the control. Therefore, the ratio of B cells *versus* myeloid cells was in favor of B cells in the Wnt3a group, and in favor of myeloid cells in the Wnt5a group (Figures 5b and c).

**Figure 6:**
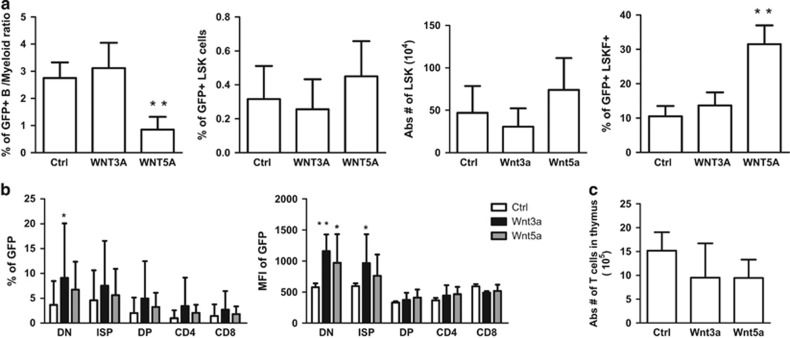
Wnt3a overexpression blocks T-cell development at early stages in the thymus. (**a**) At week 16 post transplantation, BM and Thymus are harvested and assessed by FACS Collective data depict the ratio of GFP+ B cells *versus* myeloid cells (Left), percentage and absolute number of LSKs (Middle), and percentage of LSK Flt3+^46^ in the BM. Data are mean±S.D. of five mice per group (right). (**b**) Collective data show percentage (left), or mean fluorescent intensity (MFI) (right) of GFP within each T-cell developmental stage for Ctrl group (White bar), Wnt3a group (Black bars) and Wnt5a group (gray bars). Data are mean±S.D. of five mice per group. (**c**) Bar graph depict absolute number of total thymus (thymic cellularity) within each group. Data are mean±S.D. of five mice per group. *P<0.05; **P<0.01; ***P<0.001

In the BM, the ratio of B cells *versus* myeloid cells was around threefold lower in the Wnt5a group, whereas there was no statistically significant difference between the control and Wnt3a group. MPPs (LSK Flt3+) were around threefold higher in the Wnt5a group compared with the control (Figure 6a). In the thymus, T-cell development was clearly blocked at early stages in the transduced compartment of the Wnt3a group, and to some extend with Wnt5a (as characterized by higher percentage and MFI of GFP in DN and ISP stages). However, the blocks did not affect the absolute number of mature T cells or total thymic cellularity (Figures 6b and c). Thus, Wnt3a overexpression induces B lymphopoiesis in spleen and BM whereas Wnt5a overexpression induces increased myelopoiesis in these organs.

## Discussion

Canonical Wnt signaling has a well-established role in T-cell development in the thymus; yet only a few reports deal with non-canonical Wnt signaling. A side-by-side comparison of the effects of these interacting pathways has not been performed, in contrast to for instance B-cell development^20^ and HSC biology.^37^ We therefore set out to compare the prototypical canonical Wnt ligand Wnt3a with the prototypical non-canonical Wnt ligand Wnt5a. We demonstrated that the induction of canonical Wnt signaling via Wnt3a is first, important for T-cell development, and second functions in dosage-dependent fashion. Although intermediate-to-low doses of canonical Wnt signaling is beneficial for thymopoiesis, higher doses support B lymphopoiesis *in vivo*. On the other hand, Wnt5a non-canonical Wnt signaling induces myelopoiesis *in vivo* and it does not appear to function in a strict dosage-dependent fashion. Moreover, Wnt5a signaling induces apoptosis in developing thymocytes, in line with the diminished apoptosis observed in Wnt5a-deficient thymi.

Previous studies in our laboratory and many others revealed that canonical Wnt signaling is crucial for T-cell development, and it functions in a dosage-dependent fashion.^19^ In the current study we used Wnt3a, a natural ligand of canonical Wnt signaling, and we obtained similar data. This shows that Wnt3a triggers canonical Wnt signaling via the *β*-catenin and TCF/LEF-dependent pathway in the thymus,^34^ and that it is possible to modulate canonical Wnt signaling via differential concentration of Wnt3a physiologically. It is very likely that in the thymus, developing thymocytes are exposed to different types and concentrations of Wnt ligands. It is likely that thymocytes express different FZD receptors with various binding affinity to the existing Wnt proteins within the thymic microenvironment. Previous Q-PCR data suggest that this might be the case,^21^ although experimental proof awaits the development of specific antibodies for each Frizzled receptor suitable for flow cytometry. As a result, developing thymocytes would undergo different levels of Wnt signaling owing to the accumulation of different amounts of *β*-catenin proteins in the cytoplasm.

Another possibility is that the interaction of canonical and non-canonical Wnt signaling might be important for control of *β*-catenin dosage in the cytoplasm. Sugimura *et al.*^37^ elegantly described a situation where canonical and non-canonical Wnt signaling interact with each other in the hematopoietic system. Non-canonical Wnt maintains quiescent long-term HSCs through Flamingo and Frizzled 8 receptor on HSCs. Under stress, non-canonical Wnt is attenuated and canonical Wnt is enhanced, which results in the activation of HSC. The same might be true during T-cell development in the thymus under stress, which could be a fascinating issue for future studies.

Previously, Malhorta *et al.*^20^ used a similar OP9-based Wnt3a/Wnt5 gain-of-function approach to study B lymphopoiesis. There are several differences between these two studies. (i) Their study is restricted to lympho-hematopoiesis of the BM and not thymopoiesis in the thymus.^34^ Their study was confined to *in vitro* work using FACS-purified stem cells. Importantly, using a *β*-catenin overexpression approach, Kincade and coworkers^38^ showed that committed B-cell progenitors gained myeloid lineage potential. Although we did not observe such phenomena in the T-cell lineage, Wnt3a clearly could maintain cells in an immature stage, at which thymocytes can still develop into alternative lineages. Thus, despite these differences, both studies suggest distinctive roles of Wnt family proteins during hematopoiesis and lymphopoiesis, and Wnt3a inhibits progenitor cell differentiation. However, the effect of Wnt5a on B-cell development is controversial. Malhorta *et al.*^39^ showed that it induces B lymphopoiesis, whereas Liang *et al.* showed inhibition of B-cell proliferation. We did not observe any effect of Wnt5a on B-cell development even at higher doses (data not shown) which could be due to the difference in timing, source of stem cells or concentration of Wnt proteins.

Khoo *et al.*^40^ have demonstrated that human aged HSCs have a reduced canonical Wnt signaling activity, and as a result impaired or delayed T-cell development, which is restricted to the T-cell progenitors, indicating the significance of Wnt signaling in human T-cell development during senescence. The notion that non-canonical Wnt signaling mimics murine HSC ageing has been put forward by Florian *et al.*^41^ They reported that a shift from canonical to non-canonical Wnt signaling occurs during HSC ageing. Wnt5a treatment of young HSCs induced ageing associated stem cell apolarity, and an lymphoid to myeloid differentiation skewing, which is observed normally during ageing. We performed similar experiments in which we transduced HSC with Wnt3a and Wnt5a, rather than *ex vivo* treatment. Similarly, myelopoiesis was enhanced in Wnt5a-overexpressing HSC, and B lymphopoiesis in Wnt3a-overexpressing HSC (Figures 5 and 6). Therefore, Wnt3a treatment of stem cells before transplantation could be considered a promising approach to improve lymphopoiesis.

A series of studies in the laboratory of Perreault and coworkers^30, 31, 32^ suggest that Wnt4 is a non-canonical Wnt ligand, enhances MPP expansion in the BM and ETP proliferation in the thymus, which results in increased thymic cellularity. We have also shown that Wnt5a overexpression induces MPP expansion, and DN expansion in thymus (Figure 6). However, this did not affect the total cellularity of the organs. This suggests specific roles of various Wnt family members during different stages of thymopoiesis, probably due to their differential expression throughout the thymus and/or differential responsiveness of developing thymocytes. For instance, Wnt4 has been proposed to function as a canonical Wnt in inducing FoxN1 expression in TEC,^42^ but seems to act as a non-canonical Wnt in studies aimed at improving thymic reconstitution from hematopoietic rather than thymic stromal cells.^30^

Previous studies indicate that deregulation of Wnt signaling occurs in leukemia.^43^ We have also shown that deregulation of Wnt signaling due to the absence of Tcf1, induces lymphomas in mice.^44^ Independent of our study, Yu *et al.*^11^ also reported similar results and showed similarities of Tcf1 lymphomas with human ETP-ALL cases. In addition, Martins *et al.*^45^ recently suggested that natural cell competition between young and old thymic progenitors is crucial for inhibition of T-ALL development. Therefore, thymic progenitor fitness is necessary for normal T-cell development. Our gain-of-function Wnt3a model preserves thymocytes in an immature state without inducing any malignancy. It is therefore intriguing to speculate that canonical Wnt signaling may be involved in regulating stemness of thymic stem cells.

Concluding, our work and that of others referred to above show discrete effects of Wnt3a and Wnt5a treatment on hematopoietic cells, both *in vitro* and *in vivo*. These attempts might lead to application of Wnt ligands as therapeutic candidates to improve HSC repopulation and T-cell reconstitution after SCT. However, the challenge and focus of future studies should be on determining the 'right concentration' of Wnt proteins to avoid deregulated Wnt signaling.

## Materials and Methods

Some methods are described in the supplementary data.

### Lentiviral production and transduction

Wnt3a and Wnt5a gene transfer plasmids individually cloned into the multiple cloning sites of pRRL-SFFV-IRES-GFP lentiviral vector by restriction digestion and ligation reactions. 293 T cells were transiently transfected with either the genes transfer or empty control constructs together with helper plasmids using X-TremeGENE9 transfection reagent (Roche, Basel, Switzerland). Virus containing supernatants were harvested 20 h and 40 h after transfection in Iscove's Modified Dulbecco's Medium supplemented with 10% fetal bovine serum, l-glutamin, 100 U/ml penicillin and 100 mg/ml streptomycin and used immediately for transduction, or stored at –80 °C for later use.

For transduction of OP9 stromal cells, 150 000 OP9-WT or OP9DL1 cells were seeded into a well of six-well plates with alpha MEM (Lonza, Verviers, Belgium) supplemented with 20% FCS-HI, l-glutamin and penicillin/streptomycin. The following day, OP9 cells were transduced with fresh or frozen viral supernatant containing 4 *μ*g/ml proteamine sulfate (Sigma-Aldrich, St. Louis, MO, USA). After few days of culture transduced GFP+ cells were sorted, and stable OP9-pRRL-GFP (empty vector transduced), OP9-(DL1)- W3A (Wnt3a-transduced) and OP9-(DL1)-W5A (Wnt5a-transduced) cell lines were generated. Overexpression of the genes were then confirmed by Q-PCR gene analysis. The overexpression of DL1 gene was not altered in the transduced cell lines compared with untransduced or transduced with empty vector.

### Coculture of fetal liver cells with OP9 cell lines

For different experiments various conditions of OP9 co-cultures were used as follows: OP9-WT, OP9-DL1-GFP, OP9-WT/DL1 (1 : 1), OP9-DLW3A, OP9-DLW5A, OP9-DLW3A/DL1 (1:1), OP9-DLW3A/DL1 (1 : 10) and OP9-DLW3A/DL1 (1 : 100). In all conditions 50 000 total fetal liver cells were cultured on confluent layers of OP9 cells, with AlphaMEM 10% FCS containing 50 ng/ml rmSCF, 10 ng/ml rmFlt3L and 10 ng/ml rmIL-7 (all cytokines from R&D systems, Minneapolis, MN, USA) in a well of 24-wells plate. For different purposes, cells were harvested after 6 h, 24 h, 3, 7 or 14 days of culture and stained for flow cytometric analysis.

### *In vivo* transplantation assay

Transplantation assays were performed with the CD45.1/CD45.2 system. LSK cells were sorted from CD45.2 WT fetal liver mice, overnight stimulated in stemspan in presence of Flt3-L (50 ng/ml), TPO (10 ng/ml) and SCF (100 ng/ml). Next day, sorted LSKs were transduced by means of Retronectin (Takara Bio Inc., Kusatsu, Japan) with SFFV-IRES-GFP or SFFV-Wnt3a-IRES-GFP or SFFV-Wnt5a-IRES-GFP. The viral supernatants were titrated in advance to obtain equal transduction efficiency (~50%). One day after transduction 5 × 10^3^ bulk transduced LSK cells were transplanted intravenously into lethally irradiated (8 Gy) CD45.1 (9–12 weeks) mice together with 5 × 10^5^ CD45.1 spleen support cells. Chimeras was analyzed at 4, 8 and 12 weeks after transplantation in peripheral blood, and mice were killed for analysis at 16 weeks post transplantation. Mice were considered repopulated when >1% multilineage CD45.2 cells could be detected in nucleated peripheral blood cells 3 months after transplantation.

## Abbreviations

BM: bone marrow
DLL: delta like
DN: double negative
DP: double positive
ETP: early thymic progenitor
Fzd: frizzled
GFP: green fluorescent protein
HSC: hematopoietic stem cell
ISP: immature single positive
LEF: lymphocyte enhancer factor
LSK: Lin-Sca1+c-Kit+
MPP: multipotent progenitor
SP: single positive
TCF: T-cell factor
TEC: thymic epithelial cell
