# Supplementary material for: Discrete roles of canonical and non-canonical Wnt signaling in hematopoiesis and lymphopoiesis
Source: Cell Death Dis. 2015 Nov 19;6(11):e1981–. doi: 10.1038/cddis.2015.326 (PMC4670932; doi:10.1038/cddis.2015.326)
Supplement: Supplementary Information [file cddis2015326x1.doc]

**Supplementary Figure Legends**

**Supplementary Figure 1. Comparison of OP9WT/DL and OP9 Dl1 for T cell development support**. **(A)** Total fetal liver cells were cocultured with OP9 WT (top row), OP9 DL1-GFP (middle row) or mix of OP9 WT and OP9 DL 1:1(bottom row). Cells were harvested after 4 days, 7 days and 14 days of coculture and were analysed by FACS for T cell development. The plots are pre-gated on Thy1+ LIN- markers and depict DN1-DN4 T cell developmental stages. The numbers represent percentage of each stage. **(B)** The cells were harvested 14 days and 21 days after coculture and were assessed by FACS for late stages of T cell development. The plots are pre-gated on Thy1+ marker. The numbers represent percentage of each population. Representative FACS plots of one independent experiment in triplicates are shown.

**Supplementary Figure 2. Canonical Wnt3a overexpression blocks T cell development at early stages.** Total fetal liver cells were cocultured with OP9 DL1 as control (white bar) or with OP9 DLW3A (black bars). Cells were harvested 14 days after coculture and were analysed by FACS for DN stages of T cell development. The percentage of DN stages are shown within Thy1+ LIN- populations. Error bars represent mean ± SD of two independent experiments, each in triplicates.

**Supplementary Figure 3. In vivo measurement of canonical Wnt signaling activity in thymocytes and non-T cells within the Thymus.** Activation of the canonical Wnt signaling pathway was measured using the Axin2/conductinLacZ/+ Wnt-reporter mice by FACS. (A) Quantification of frequency, and (B) the mean fluorescence intensity (MFI) of the LacZ+ populations for each subset in the thymus are depicted. Littermate mice not carrying the reporter transgene (Axin2/Conductin+/+) were used to define the LacZ+ population. For each subset, MFI of the LacZ+ population was normalized for the MFI of corresponding LacZ- population (MFI LacZ+/MFI LacZ-) in order to correct for differences in background staining between different hematopoietic populations. Data represent results from three Axin2/conductinLacZ/+ mice and two Axin2/Conductin+/+ control mice, from one independent experiment. Error bars represent mean ± SD.


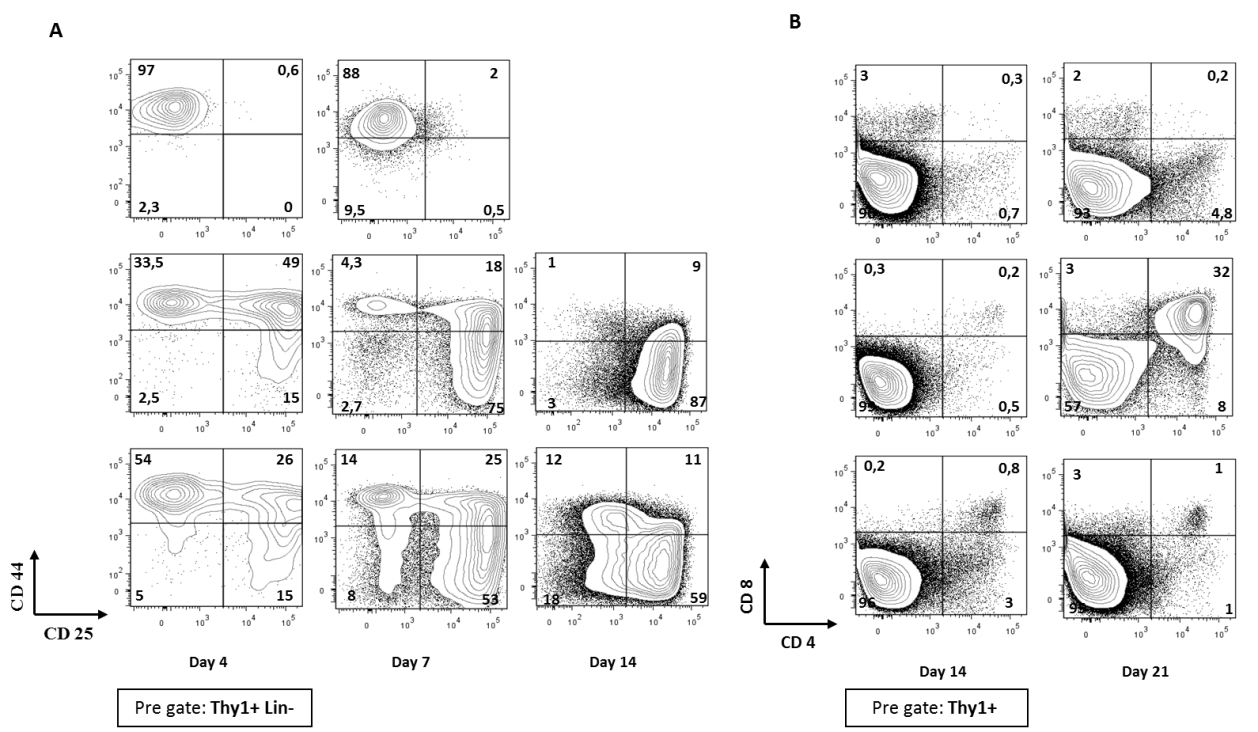


**Supplementary figure 1A-B . Famili, F et al. Discrete Roles of Canonical and Non-canonical Wnt Signalling in Hematopoiesis and Lymphopoiesis**


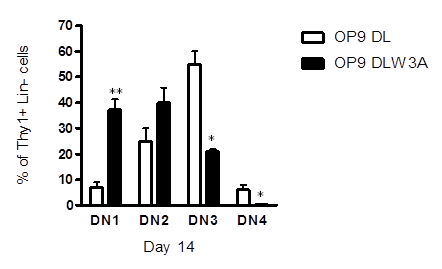


**Supplementary figure 2 . Famili, F et al. Discrete Roles of Canonical and Non-canonical Wnt Signalling in Hematopoiesis and Lymphopoiesis**


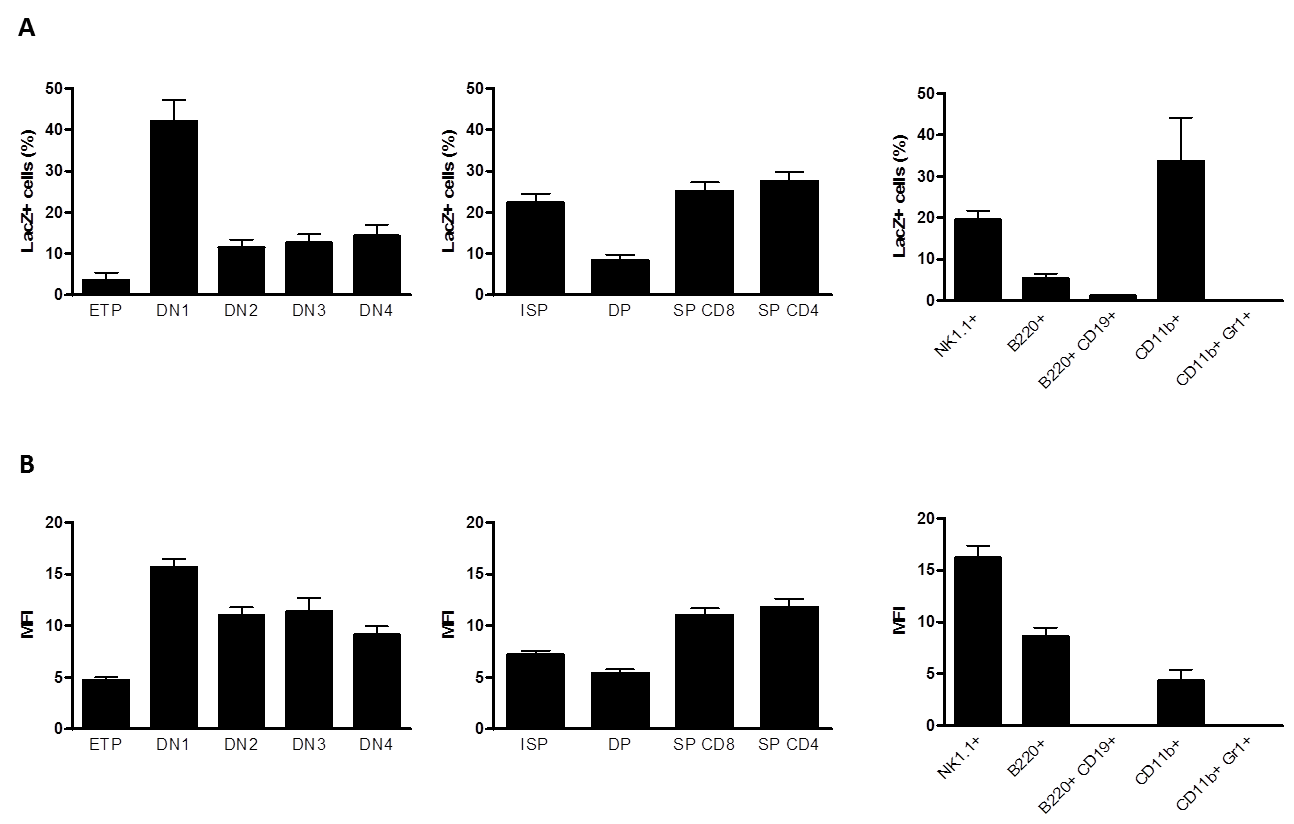


**Supplementary figure 3A-B . Famili, F et al. Discrete Roles of Canonical and Non-canonical Wnt Signalling in Hematopoiesis and Lymphopoiesis**

**Supplemental Methods**

**Mice**

Mice were bred and maintained in the animal facilities of Leiden University Medical Center, in accordance with legal regulations in The Netherlands and with the approval of the Dutch animal ethical committee. C57Bl/6-CD45.1 (Ly5.1) and C57Bl/6-CD45.2 (Ly5.2) mice were obtained from the Jackson Laboratory, and Conductin (Axin2)-LacZ mice were kindly provided by B. Jerchow and W. Birchmeier (Max Delbruck Center for Molecular Medicine, Berlin, Germany) and have been described previously ^35^.

**Flow Cytometry**

The following antibodies were obtained from BD Biosciences (San Diego, CA): anti-CD3-APC (145-2C11), anti-CD4-PeCy7 (RM4-5), anti-CD8-PerCP(53-6.7), anti-CD25-PE (PC61), anti- CD44-APC-Cy7 (IM7), anti-cKit-PeCy7 (2B8), anti CD11b-PE (M1/70), anti CD19-APC (ID3) . For Lineage depletion these markers were used: CD3 (145-2C11), CD4 (L3T4), CD8 (53-6.7), CD11b (M1/70), Gr1 (RB6-8C5), B220 (Ra3-6B2), Ter119 (Ly76) and Nk1.1 (PK136) biotin and subsequently were stained with streptavidin eFluor 450 (48-4317) from eBioscience. The following antibodies were also purchased from eBioscience: CD45.1-PE-Cy7 (A20), CD45.2 Alexa Fluor 780 (104), B220 PE-Cy7 (RA3-6B2), Thy1.2 APC (53-2.1), Gr1 eFluor 450 (RB6-8C5) and Sca1 PE-Cy7 (D7). Cells were stained in Fluorescenceactivated cell sorter (FACS) buffer (PBS, 2% bovine serum albumin, 0.1% sodium azide) for 30 min at 4 °C. Ultimately, Cells were washed and measured either on a Canto I, or an Aria (BD Biosciences). For apoptosis analysis cells were stained with 7AAD/AnnexinV (BD Bioscience). Data were analyzed using FlowJo software (Tree Star, Ashland, OR, USA).

Intracellular β-galactosidase activity was measured by staining cells with 2 mM fluorescein di-b-D-galactopyranoside (FDG) substrate (Molecular Probes). FDG was loaded into the cells by hypotonic shock at 37 °C for 1 min, prior to cell surface antibody staining. The β-galactosidase reaction was stopped with 1 mM phenylethyl b-D-thiogalactopyranoside (PETG, from Molecular Probes) after 3 hours as described before and optimized for use in thymocytes ^36^.

**Gene expression analysis**

Total RNA was extracted using Qiagen RNeasy mini or micro columns. One ug of total RNA was used as a template for cDNA synthesis, using Superscript III reverse transcriptase (Invitrogen, Carlsbad, CA, USA), Oligo dT, and random hexamer primers. The RQPCR reaction was performed using TaqMan Universal mastermix (Applied biosystems, Foster City, CA, USA) and was run on a PRISM 7700 sequence detection system containing a 96-well thermal cycler (Applied Biosystems). The following primers were used in combination with FAM-labelled probes from the universal probe library (Roche): Delta Like-1 forward primer: 5’- GGGGAGAGAGGGGAGAAGAT - 3’; reverse primer: 5’- ACAGCCTGGCAGACAAATG -3’; Wnt3a forward primer: 5’- CTTAGTGCTCTGCAGCCTGA -3’; reverse primer: 5’- GAGTGCTCAGAGAGGAGTACTGG -3’; Wnt5a forward primer: 5’- ATGAAGCAGGCCGTAGGAC -3’; reverser primer: 5’- CTTCTCCTTGAGGGCATCG -3’; Axin2 forward primer: 5’-GCAGGAGCCTCACCCTTC-3’; reverse primer: 5’- TGCCAGTTTCTTTGGCTCTT-3’. RQ-PCR results were normalized to Abl expression in the same sample: forward primer: 5’-TGGAGATAACACTCTAAGCATAACTAAAGGT-3’; reverse primer: 5’-GATGTAGTTGCTTGGGACCCA-3’; and probe: 5’-FAM-CCATTTTTGGTTTGGGCTTCACACCATT- NFQ-3’.

**Statistical analysis**

Statistical analysis was performed using the Mann–Whitney U test (Prism GraphPad Software, San Diego, CA, USA). P < 0.05 was considered statistically significant. Asterisks indicate statistical significance as follows: * P < 0.05, and ** P < 0.01. Otherwise P values are mentioned.
